# Potential of ferritin 2 as an antigen for the development of a universal vaccine for avian mites, poultry red mites, tropical fowl mites, and northern fowl mites

**DOI:** 10.3389/fvets.2023.1182930

**Published:** 2023-04-17

**Authors:** Shwe Yee Win, Shiro Murata, Sotaro Fujisawa, Hikari Seo, Jumpei Sato, Yoshinosuke Motai, Takumi Sato, Eiji Oishi, Akira Taneno, Lat Lat Htun, Saw Bawm, Tomohiro Okagawa, Naoya Maekawa, Satoru Konnai, Kazuhiko Ohashi

**Affiliations:** ^1^Laboratory of Infectious Diseases, Department of Disease Control, Faculty of Veterinary Medicine, Hokkaido University, Sapporo, Japan; ^2^Department of Advanced Pharmaceutics, Faculty of Veterinary Medicine, Hokkaido University, Sapporo, Japan; ^3^Vaxxinova Japan K.K., Tokyo, Japan; ^4^Department of Pharmacology and Parasitology, University of Veterinary Science, Nay Pyi Taw, Myanmar; ^5^Department of Livestock and Aquaculture Research, Ministry of Agriculture, Livestock and Irrigation, Nay Pyi Taw, Myanmar; ^6^International Affairs Office, Faculty of Veterinary Medicine, Hokkaido University, Sapporo, Japan

**Keywords:** poultry red mite, tropical fowl mite, northern fowl mite, ferritin 2, vaccine

## Abstract

**Introduction:**

Poultry red mites (PRMs, *Dermanyssus gallinae*), blood-sucking ectoparasites, are a threat to the poultry industry because of reduced production caused by infestation. In addition, tropical fowl mites (TFMs, *Ornithonyssus bursa*) and northern fowl mites (NFMs, *Ornithonyssus sylviarum*) are hematophagous, distributed in various regions, genetically and morphologically close to PRMs, and cause similar problems to the poultry industry. Vaccine approaches have been studied for PRM control, and several molecules have been identified in PRMs as candidates for effective vaccine antigens. The development of an anti-PRM vaccine as a universal vaccine with broad efficacy against avian mites could improve the productivity of poultry farms worldwide. Molecules that are highly conserved among avian mites and have critical functions in the physiology and growth of mites could be ideal antigen candidates for the development of universal vaccines. Ferritin 2 (FER2), an iron-binding protein, is critical for the reproduction and survival of PRMs and has been reported as a useful vaccine antigen for the control of PRMs and a candidate for the universal vaccine antigen in some tick species.

**Method and results:**

Herein, we identified and characterized FER2 in TFMs and NFM. Compared with the sequence of PRM, the ferroxidase centers of the heavy chain subunits were conserved in FER2 of TFMs and NFMs. Phylogenetic analysis revealed that FER2 belongs to clusters of secretory ferritins of mites and other arthropods. Recombinant FER2 (rFER2) proteins from PRMs, TFMs, and NFMs exhibited iron-binding abilities. Immunization with each rFER2 induced strong antibody responses in chickens, and each immune plasma cross-reacted with rFER2 from different mites. Moreover, mortality rates of PRMs fed with immune plasma against rFER2 from TFMs or NFMs, in addition to PRMs, were higher than those of control plasma.

**Discussion:**

rFER2 from each avian mite exhibited anti-PRM effects. This data suggests that it has the potential to be used as an antigen candidate for a universal vaccine against avian mites. Further studies are needed to access the usefulness of FER2 as a universal vaccine for the control of avian mites.

## Introduction

The poultry red mite (PRM, *Dermanyssus gallinae*), is a harmful ectoparasite for poultry and is prevalent worldwide (1). Blood feeding by PRMs leads to reduced animal welfare and serious economic losses on poultry farms. Tropical fowl mites (TFMs, *Ornithonyssus bursa*) and northern fowl mites (NFMs, *Ornithonyssus sylviarum*) are ectoparasites of untamed birds (2), and are widely spread as key pests of poultry because of their introduction to farms *via* wild birds (3). Once invaded, these mites can persist long-term in farm facilities and poultry (4). PRMs feed on the host blood for a short period, mainly at night, and leave the hosts after blood feeding, residing in cracks and crevices for the rest of the time (5). In contrast, TFMs and NFMs are parasitic on the hosts throughout their life cycle (6). PRMs are widespread worldwide, with >46% of poultry farms in China and Japan (7) and 90% of the layer industry in Europe being affected (8). TFMs are cosmopolitan in tropical and subtropical countries (7, 9). Although the issues caused by NFMs are not uniform across the world, they are included as key pests in the poultry industry in North America, Brazil, Australia, and Asia (7, 10–12).

The success of acaricide treatment is hindered by the selection of mites resistant to acaricides owing to prolonged or improper application on farms (13). Because of the diminished efficacy of commercially available acaricides, they may have short residual action on mites. Consequently, the subclinical stages of mites or their eggs could enable a cycle of mite repopulation on farms (8). Some natural products, such as essential oils and plant derivatives, have been studied for their non-chemical acaricidal effects (14, 15). However, they may contain some active ingredients and may be harmful to humans and animals (16). Currently, vaccine-based control strategies are considered promising. Several recombinant protein-based anti-PRM vaccines have been reported by our research group (17–21) and other groups (22–25). Immunization with anti-PRM vaccines induces antigen-specific immunoglobulin (Ig)-Y in chickens, leading to reduced PRM survival in *in vivo* or *in vitro* studies. However, vaccine efficacy has not been sufficient for practical use in farms (23, 26). Therefore, the search for more effective antigens against PRMs is required, and antigens with broad protective efficacies across avian mites are more suitable for reducing the economic losses on poultry farms in various areas than those with limited protective efficacies.

For research on anti-tick vaccine development, molecules that are in direct contact with the host during blood feeding and are required to create an environment for blood feeding (exposed antigens) and molecules that are not exposed to the host and have essential physiological functions for the mites (concealed antigens) are considered vaccine candidates (27). Similar to the strategy of anti-tick vaccines, the development of anti-mite vaccines should focus on molecules involved in the key physiological functions of avian mites. As blood meal is the nutrient source for blood-sucking ectoparasites, including avian mites, the molecules involved in blood digestion and acquisition of essential nutrients could be suitable candidates for vaccine antigens with a broad spectrum across hematophagous avian mites. Iron is an essential nutrient for blood-feeding ectoparasites; however, the excessive presence of iron could be toxic. Therefore, iron homeostasis must be precisely controlled in the blood-feeding ectoparasites. Ferritin (FER), an iron-binding protein, is involved in iron homeostasis in most organisms (28). Two types of FER, FER1 and FER2, have been identified in ticks (29, 30), and both are fundamentally involved in blood feeding, reproduction, iron transport, and antioxidant defense (31). FER1 plays a role in intracellular iron storage and serves as an antioxidant by sequestering excess intracellular iron, whereas FER2 is a secreted ferritin that plays a role in the transportation of iron to peripheral tissues (29). In PRMs, two FERs have been identified, and their detrimental effects on survival, reproduction, and blood digestion have been demonstrated by RNA interference (RNAi) assays. Furthermore, both ferritins showed acaricidal potentials as vaccine antigens; importantly, the survival rate of PRMs fed with the plasma of chickens immunized with rFER2 (rDg-FER-1 in the original study) was significantly reduced compared with those of the rFER1-immunized group (rDgFER-2 in the original study) (25). Therefore, the potential of FER2 as a vaccine antigen has induced our interest in the development of a universal vaccine with broad-spectrum efficacy across mite species.

Development of a universal vaccine is of significant importance in veterinary practice. The successful application of universal vaccines offers cost-effectiveness by reducing the number of vaccine antigens, because there is no requirement to prepare species-specific antigens. A vaccine using Bm86, which has the potential to cross-react with different species of ticks, has been highlighted as a benefit to the livestock industry (32, 33). Additionally, glutathione S-transferase (34, 35), FER2 (27, 36), and subolesin (37) have been reported as vaccine antigen candidates for cross-species universal vaccines. However, the number of tick species for which the vaccine showed efficacy is limited and its effectiveness at various developmental stages has not yet been established (38). As for the control of avian mites, the development of vaccines against PRMs has progressed, whereas there are no reports of vaccine development against other avian mites, such as TFMs and NFMs, to the best of our knowledge. Therefore, the development of a cross-protective vaccine could be a sustainable management strategy for avian hematophagous mites on poultry farms, and it may save the economic losses on poultry farms and improve the cost-effectiveness of commercial production. Thus, in the present study, we aimed to investigate the potential of FER2 as a common antigen for developing a universal vaccine for avian mites. We identified the *FER2* genes from TFMs and NFMs, evaluated the iron-binding ability of each rFER2, and investigated the cross-reactivity of immune plasmas against each rFER2 with the rFER2 of different mites to assess the acaricidal potential of immunization with rFER2 for avian mites. Additionally, the acaricidal effects on PRMs by the immune plasmas from TFMs and NFMs were assessed.

## Material and methods

###  Sample availability, RNA extraction, and complementary DNA synthesis

PRMs were collected into a TubeSpin Bioreactor 600 bottle (TPP Techno Plastic Products AG, Trasadingen, Switzerland) from egg-laying farms contaminated with PRMs in Japan and transferred to the laboratory at 4°C. PRMs were kept at 25°C for a week without blood feeding, designated as starved PRMs, and stored at 5°C for further use. TFMs and NFMs, collected in the Republic of the Union of Myanmar (Burma), which were morphologically and genetically characterized in a previous study (39), were used for analysis in this experiment. Total RNA was extracted using TRIzol reagent (Invitrogen, Carlsbad, CA, USA), according to the manufacturer's instructions. Complementary DNA (cDNA) was synthesized from 1 μg of isolated RNA using PrimeScript Reverse Transcriptase (Takara Bio Inc., Shiga, Japan) and 200 pmol of oligo (dT)18 primer (Hokkaido System Science, Hokkaido, Japan). The synthesized cDNAs was treated with DNase I (Invitrogen) to remove unwanted DNA.

###  Identification of *ferritin 2* genes from TFMs and NFMs

To determine the open reading frames (ORFs) of *FER2* from TFMs and NFMs, partial segments were amplified using primers designed based on the conserved region of *FER2* from *Dermanyssus gallinae* (HZ459285) and *Varroa destructor* (XM022808086). Primers used in this study are listed in Supplementary Table 1. The amplified fragments were cloned into a pMD20 vector (Takara Bio Inc.). Nucleotide sequences were analyzed using the CEQ GeXP automated sequencer (Beckman Coulter Inc., Brea, CA, USA). The primers used for the 3′ and 5′ RACE polymerase chain reaction (PCR) amplifications were designed based on the partial sequences of *FER2*. We conducted 3′ and 5′ RACE PCR using the RACE system (Invitrogen) according to the manufacturer's instructions. The PCR products were separated by agarose gel electrophoresis, purified, and cloned into the pGEMT-Easy vector (Promega, Madison, WI, USA).

###  Genetic characterization of *FER2*

Homologies of *FER2* genes of TFMs and NFMs with reported sequences (Supplementary Table 2) from the National Center for Biotechnology Information gene bank were compared using the Basic Local Alignment Search Tool program. We constructed a phylogenetic tree using the nucleotide sequences of the *FER2* genes of arthropods, including other mites, ticks, and chickens, and their sequences, using MEGA X software (40). These sequences were aligned using the MUSCLE (codon) option. A maximum-likelihood phylogenetic tree was constructed using the same software with 1,000 bootstrap replicates and a discrete gamma distribution (+G) to improve the tree topology.

###  Expression and purification of recombinant ferritin 2 proteins

The coding regions of *FER2* genes were amplified with Taq polymerase (Takara Bio Inc.) using specific primers containing the sites of NdeI and XhoI for introduction into the pET19b vector (Merck & Co., Inc., Rahway, NJ, USA) (Supplementary Table 1). The amplified fragments were cloned into the pET19b vector (Merck) and transformed into *Escherichia coli* strain Rosetta-gami B (DE3, pLysS) (Merck). We generated N-terminal His-tagged rFER2 proteins of PRMs, TFMs, and NFMs using the *E. coli* expression system, termed as rFER2 PRM, rFER2 TFM, and rFER2 NFM, respectively. For rFER2 PRM generation, the reference sequence of FER2 (HZ459284) from Japan was used. Recombinant protein expression and purification were performed according to the manufacturer's instructions. The cell pellets were fractionated with BugBuster solution (Merck), and the insoluble fractions were solubilized in the buffer containing 0.3% N-lauroylsarcosine, 50-mM N-cyclohexyl-3-aminopropanesulfonic acid (CAPS) (Merck) (pH 11.0). Recombinant proteins were purified from insoluble fractions using Ni Sepharose™ 6 Fast Flow resin (GE Healthcare, Chicago, IL, USA) according to the manufacturer's instructions. The recombinant proteins were eluted with 0.3% N-lauroylsarcosine, 50-mM CAPS (pH 11.0) containing 250-mM imidazole (Nacalai Tesque, Tokyo, Japan). The purified FER2 proteins were refolded by dialysis against a 10-mM Tris-HCL (pH 8.5) buffer containing 0.1-mM DL-dithiothreitol (Merck) at 4°C overnight. The purity of the recombinant proteins was analyzed using 13% sodium dodecyl sulfate-polyacrylamide gel electrophoresis (SDS-PAGE) and stained with Coomassie brilliant blue (FUJIFILM Wako Pure Chemical Corporation, Osaka, Japan). The concentration of recombinant proteins was determined using the Pierce™ BCA Protein Assay Kit (Thermo Fisher Scientific, Waltham, MA, USA) with bovine serum albumin (BSA) as the standard, according to the manufacturer's instructions.

###  Iron binding assay

A ferrozine-based iron-binding assay was performed to analyze the iron-binding ability of rFER2 proteins (31). Different concentrations of rFER2 were dissolved in 954 μL of double distilled water and mixed with 20 μL of 1-M HEPES (pH 7.0) and 1 μL of 40-mM Fe_2_(NH_4_)_2_(SO_4_)_2_. After incubation at 30°C for 30 min, 20 μL of 10-mM ferrozine (Sigma-Aldrich, St. Louis, MO, USA) was added and incubated for 30 min. Each mixture (300 μL) was transferred to 3 wells of a microplate. The absorbance was measured at 570 nm using a microplate reader (Corona Electric, Hitachinaka, Japan). For the analyses, we used 2.5, 5, and 10 μg/mL of each rFER2 protein, apoferritin from equine spleen (Sigma-Aldrich) as the positive control, and BSA (Merck) as the negative control. All values were indicated as means, and error bars indicate standard deviations.

###  Immunization of chickens with rFER2 proteins

Sixteen chickens were randomly allocated to four groups: rFER2 PRM, rFER2 TFM, and rFER2 NFM immunization groups and the control group. To generate immune plasma, four chickens per group were subcutaneously immunized with 20 μg of each rFER2 mixed with the Freund incomplete adjuvant (FUJIFILM Wako Pure Chemical Corporation) at 3 weeks of age. The chickens were boosted at 3 weeks after the first immunization with 20 μg of recombinant proteins with the same adjuvant. As the control, four chickens were immunized with phosphate-buffered saline (PBS) and mixed with the same adjuvant. Three weeks after the second immunization, heparinized blood was collected from each chicken, and immune plasma was isolated.

###  Western blotting

Western blotting was performed to ascertain the antibody responses to each rFER2 vaccination and analyze the cross-reactivity of each immune plasma. The rFER2 proteins were electrophoresed on 13% SDS-PAGE gel and transferred onto polyvinylidene difluoride membranes (Merck). The membranes were blocked with 3% skim milk at 4°C overnight. Membranes were incubated with isolated immune plasma (1:1,000) at 25°C for 1 h and washed 3 times with PBST. The membranes were incubated with an anti-chicken IgY peroxidase rabbit antibody (1:10,000) (Sigma-Aldrich) at 25°C for 1 h and washed 3 times with PBST. Finally, the signal was detected using the Immobilon Western Chemiluminescent HRP Substrate (Merck).

###  Enzyme-linked immunosorbent assay

The enzyme-linked immunosorbent assay (ELISA) was performed to determine the antibody titers of the immune plasma against each rFER2. Briefly, 100 ng/well of each rFER2 protein was coated onto an ELISA plate (Sumitomo Bakelite Co. Ltd., Tokyo, Japan) with a carbon-bicarbonate buffer (pH 9.8) at 4°C overnight. The plate was washed 3 times with PBST and blocked with PBST-containing 1% BSA at 37°C for 2 h. After blocking, diluted immune plasma was added to each well and incubated at 25°C for 30 min. The wells were then washed with PBST 5 times and incubated with anti-chicken IgY[IgG](H+L)-HRP (goat; Bethyl Laboratories, Inc., Montgomery, TX, USA) at 37°C for 1 h. The reaction was detected by adding TMB One Component HRP Microwell Substrate (Bethyl Laboratories, Inc.) to each well, followed by incubation at 37°C for 15 min. After adding 100 μL of 0.18 M H_2_SO_4_ to stop the reaction, the absorbance was measured at 450 nm in a microplate reader (Corona Electric).

### *In vitro* feeding assay

Fresh heparinized blood was collected from healthy chickens maintained at the Field Science Center for Northern Biosphere, Hokkaido University and incubated at 40°C before use. Plasma samples from chickens unimmunized or immunized with each rFER2 were pooled separately for each group. Plasma from heparinized fresh blood was replaced with each pool of plasma. Starved PRMs of mixed developmental stages were collected into *in vitro* feeding devices (17), and blood feeding was performed for 4 h at 40°C in dark and humid conditions with shaking at 100 rpm. Only blood-fed PRMs were collected using Pasteur pipettes and maintained at 25°C in 60% humidity throughout the monitoring period. The number of dead PRMs in each group was counted daily for a week, and the acaricidal effects of immune plasma against rFER2 were evaluated based on the survival rate of PRMs (the number of dead PRMs / the number of blood-fed PRMs).

### Statistical analysis

In the iron-binding assay, statistical comparisons were performed using the Kruskal–Wallis test and the Steel–Dwass comparison test; asterisks indicate significant differences (^*^P < 0.05 and ^**^P < 0.01). To compare PRM mortality between the immunized and control groups after *in vitro* feeding, we generated Kaplan–Meier curves and performed a log-rank test with Bonferroni corrections on multiple comparisons. Additionally, the Fisher exact test was performed to compare the mortality of PRMs between the groups on each day. All statistical analyses were performed using EZR, an easy-to-use software based on R and R commander (41). Moreover, the odds ratios and 95% confidence intervals were calculated. *P* < 0.01 for log-rank test and *P* < 0.05 for Fisher exact test were considered statistically significant.

## Results

###  Identification and genetic characterization of the *FER2* genes in TFMs and NFMs

**Figure 1:**
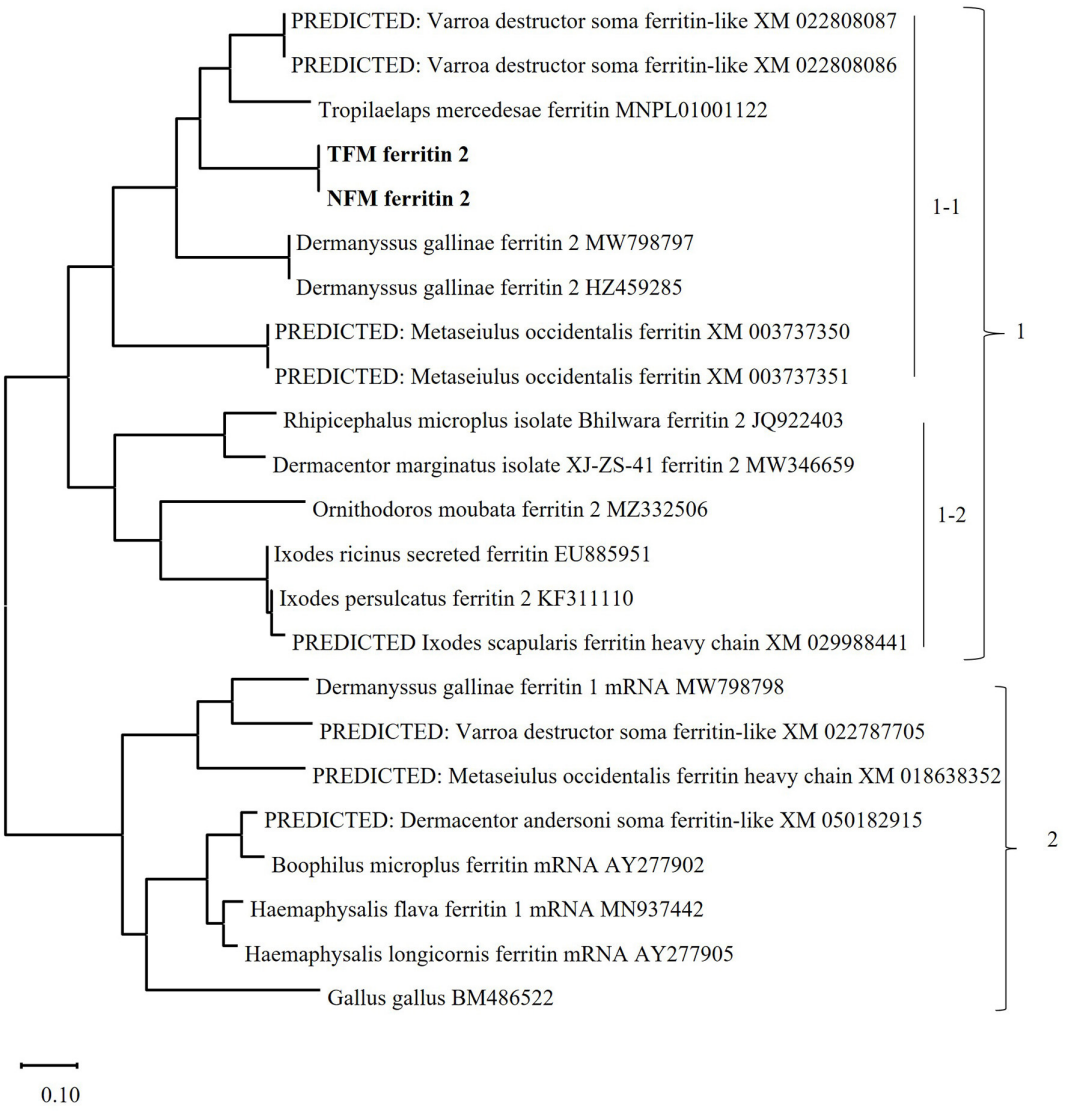
Phylogenetic analysis of the *ferritin* (*FER*) genes from poultry red mites (PRM), tropical fowl mite (TFM), northern fowl mite (NFM), arthropods, including other mites and ticks, and chickens. The phylogenetic tree was constructed using the maximum-likelihood method with MEGA X software. The numbers on the right indicate the clusters. Cluster 1 includes secretory types of *FER2* genes, and is divided into 2 subclusters. Subcluster 1-1: The secretory types of *FER2* genes of mites are classified into this subcluster, and the *FER2* genes of PRMs, TFMs (bold), and NFMs (bold) belong to this cluster. Subcluster 1-2: This cluster consist of secretory types of *FER2* genes from ticks. Cluster 2: The intracellular *FER* genes of mites, ticks, and chickens are classified into this cluster.

The nucleotide sequences of the ORFs of *FER2* genes from TFMs (LC752110) and NFMs (LC752111) consisted of 588 bp with 195 amino acids and signal peptides at positions 1–17. The *FER2* genes of TFMs and NFMs showed 99.0% homology with each other and 65.0% homology with those of PRMs (Supplementary Figure 1; Table 1). In addition, the ferroxidase centers, which are iron-binding sites for the oxidation of Fe(II) in heavy-chain (H) ferritin, were completely conserved with those of PRMs. To genetically characterize the *FER2* genes of TFMs and NFMs, we constructed a phylogenetic tree using the sequences of *FER* genes from mites, including PRMs, ticks, and chickens (Figure 1). The *FER2* genes of TFMs and NFMs were distinctly classified into cluster 1, consisting of secreted *ferritin* (*FER2*) genes, and were most closely related to the secreted *ferritin* genes of PRM, *Varroa destructor*, and *Tropilaelaps mercedesae* in subcluster 1-1 consisting of secreted *ferritin* genes of mites. The subcluster 1-2 included secreted *ferritin* genes in ticks. Cluster 2 included intracellular *ferritin* (*FER1*) genes from PRMs and other mites, ticks, and chicken. Thus, *FER2* genes of mites, including TFMs and NFMs, clearly belong to the secretory type of ferritins.

**Table 1:** Comparison of sequences of ferritin 2 from poultry red mites, northern fowl mites, and tropical fowl mites.

		**Amino acid sequence (%)**		
		**PRM**	**TFM**	**NFM**
Nucleotide sequence (%)	PRM	–	65.0	66.0
	TFM	67.0	–	99.0
	NFM	68.0	99.0	–

PRM, poultry red mite; NFM, northern fowl mite; TFM, tropical fowl mite.

###  Iron binding ability of FER2 proteins of PRMs, TFMs, and NFMs

**Figure 2:**
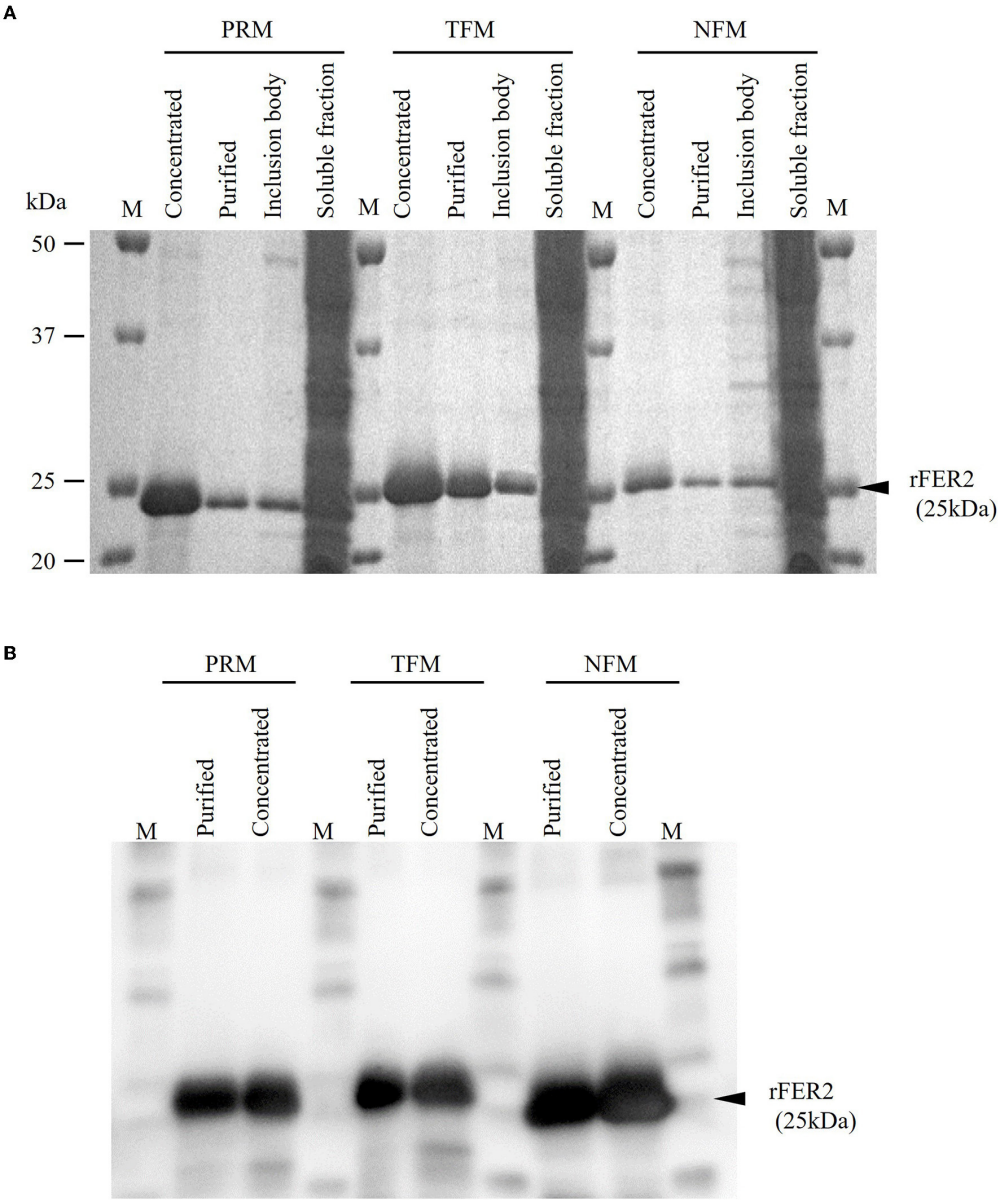
Expression and purification of recombinant ferritin 2 (rFER2) proteins. The ferritin 2 from poultry red mites (PRMs), tropical fowl mite (TFMs), and northern fowl mites (NFMs) were expressed and purified as recombinant proteins fused with histidine tag, and named as rFER2 PRM, rFER2 TFM, and rFER2 NFM, respectively. rFER2 from each mite was expressed in *Escherichia coli* and purified from the inclusion body fraction by affinity chromatography. The purity of rFER2 was confirmed by sodium dodecyl sulfate-polyacrylamide gel electrophoresis **(A)** and Western blotting **(B)**. M, Marker (Precision Plus Protein™ All Blue Prestained Protein Standards; Bio-Rad, Hercules, CA, USA).

**Figure 3:**
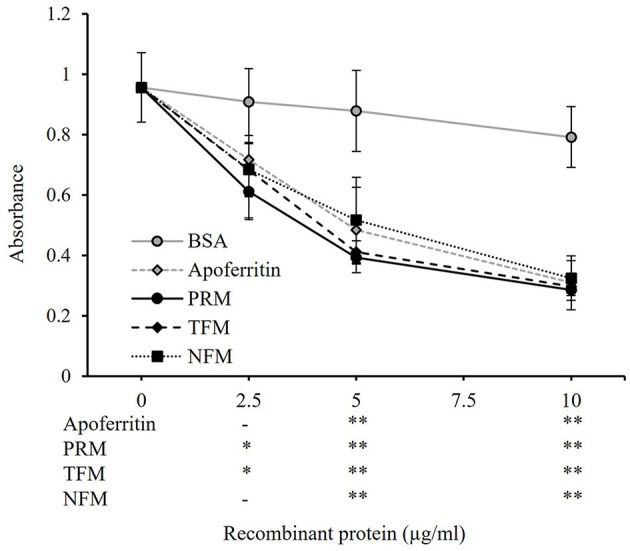
Iron-binding ability of recombinant ferritin 2 (rFER2) proteins. The iron-binding ability of rFER2 proteins from poultry red mite, tropical fowl mite, and northern fowl mite was assessed by a ferrozine-based colorimetric assay using different concentrations of each rFER2. Ferrozine was used as an indicator agent. Bovine serum albumin and horse apoferritin were used as the negative and positive controls, respectively. The *x*-axis indicates the amounts of rFER2 proteins used in this assay. Error bars indicate standard deviations. Statistical differences are shown as a comparison to bovine serum albumin (BSA). Asterisks indicate significant differences (^*^P < 0.05 and ^**^P < 0.01).

The whole regions of FER2 from PRMs, TFMs, and NFMs, excluding the signal peptides, were generated as recombinant proteins fused with His-tag, termed as rFER2 TFM, rFER2 NFM, and rFER2 PRM. The recombinant proteins were purified from the insoluble fractions by affinity chromatography, and their purities were confirmed by SDS-PAGE and Western blotting (Figures 2A, B). To assess whether each rFER2 has iron-binding ability, we conducted a ferrozine-based colorimetric assay. The absorbance of rFER2 proteins in PRMs, TFMs, and NFMs decreased in a dose-dependent manner, similar to that of apoferritin, the positive control (Figure 3). Thus, these results showed that rFER2 proteins of PRMs, TFMs, and NFMs have iron-binding abilities.

###  Cross-reactivities of antibodies produced by the immunization with rFER2

**Figure 4:**
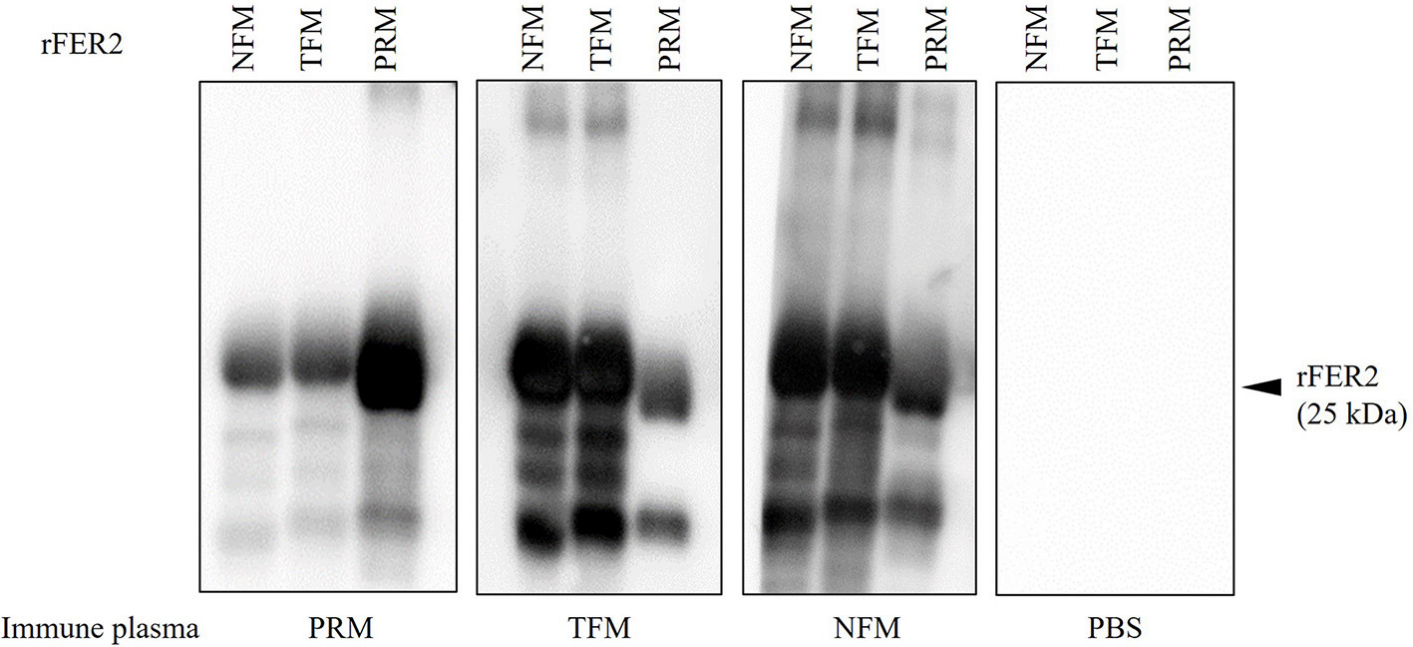
The production of specific antibodies in the plasma from chickens immunized with recombinant ferritin 2 (rFER2) proteins. Four chickens per group were immunized with rFER2, separately. The plasmas were isolated from each immunized chicken, and the production of antibodies specific to rFER2 in the plasma were detected by Western blotting. The cross-reactivities of immune plasmas with each rFER2 were tested by Western blotting. The arrowhead indicates the predicted molecular weight of rFER2 (~25 kDa).

To examine the potential of rFER2 as a universal vaccine antigen against avian mites, immune plasma was isolated from chickens immunized with each rFER2. As shown in Table 2, increased production of antibodies in the immunized groups was confirmed. Western blotting revealed the presence of chicken IgY specific to rFER2 in the immune plasma (Figure 4). Additionally, the immune plasma against each rFER2 reacted with all rFER2 proteins, including those from different mites. However, the intensity of the signals was slightly different when the reactivity of immune plasma against rFER2 PRM was compared between rFER2 PRM and those of TFMs and NFMs, and vice versa. These results suggest that rFER2s have potential as common vaccine antigens in avian mites.

**Table 2:** Antibody titers in plasma samples from chickens immunized with recombinant ferritin 2.

**Group**	**Chicken**	**Antibody titer**
Control	C1	< 2,000
	C2	< 2,000
	C3	< 2,000
	C4	< 2,000
Immunized- rFER2 PRM	PRM1	32,000
	PRM2	128,000
	PRM3	128,000
	PRM4	64,000
Immunized- rFER2 TFM	TFM1	128,000
	TFM2	128,000
	TFM3	64,000
	TFM4	64,000
Immunized- rFER2 NFM	NFM1	64,000
	NFM2	32,000
	NFM3	16,000
	NFM4	32,000

PRM, poultry red mite; NFM, northern fowl mite; TFM, tropical fowl mite; rFER2, recombinant ferritin 2.

###  Assessment of acaricidal activity of the plasmas from chickens immunized with each rFER2

**Figure 5:**
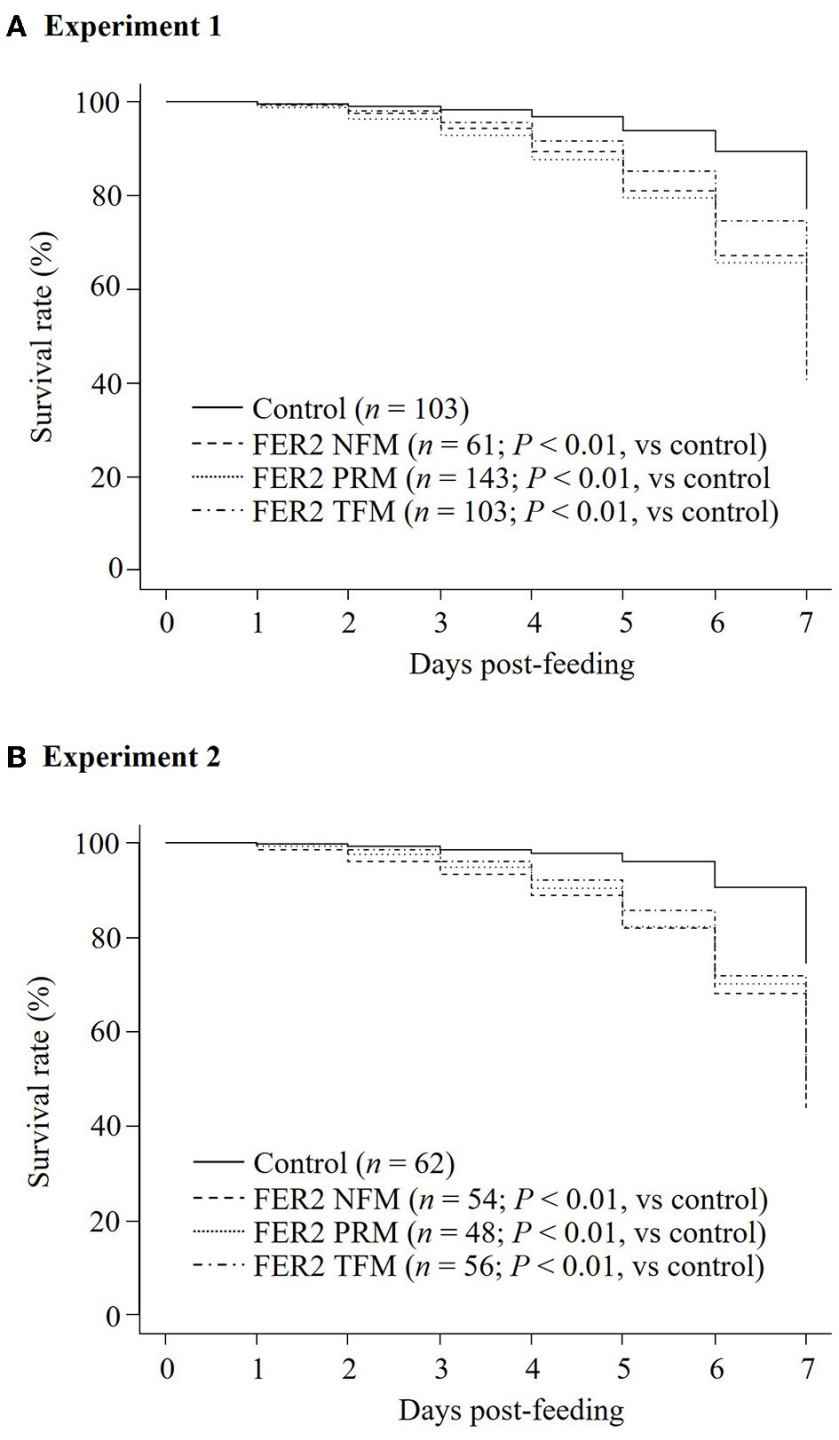
Assessment of the acaricidal potential of plasma obtained from chickens immunized with recombinant ferritin 2 (rFER2) proteins. Artificial blood feeding to poultry red mite (PRMs) was performed by the *in vitro* feeding assay. The survival rates of PRMs fed with immune plasmas or control plasma were monitored daily for a week. The *in vitro* feeding assays were performed 2 times. The total number of PRMs used in this study is as follows: **(A)** experiment 1: fed with immune plasma: *n* = 143 (rFER2 PRM), *n* = 103 [rFER2 tropical fowl mite (TFM)], and *n* = 61 [rFER2 northern fowl mite (NFM)] and fed with control plasma: *n* = 103; **(B)** experiment 2: fed with immune plasma: *n* = 48 (rFER2 PRM), *n* = 56 (rFER2 TFM), and *n* = 54 (rFER2 NFM) and fed with control plasma: *n* = 62. The number of dead PRMs were recorded and plotted on the graph to generate Kaplan–Meier survival curves. Statistical analysis was performed using a log-rank test with Bonferroni corrections on multiple comparisons. *P* < 0.01 was considered statistically significant between each immunized and control group.

To assess the acaricidal effects of each FER2, we performed *in vitro* feeding assays and monitored the mortality of PRMs fed immune plasmas against each FER2. In this study, we used PRMs for *in vitro* feeding assays because of the limited distribution of NFMs and the absence of TFMs in Japan. To examine the acaricidal effects, we compared mortality between the immunized and control groups using the Fisher exact and log-rank tests. In this study, we used pooled plasma of chickens from each immunized group and found that the antibody titer of each group was different. According to a previous report, the acaricidal effects could depend on the antibody titer (21). Therefore, we only compared the mortality between the control group and each immunized group. The *in vitro* feeding assays were performed twice. In experiment 1, the mortality rate of PRMs reached 40.55, 32.03, and 39.34% in immunized groups of rFER2 PRM, rFER2 TFM, and rFER2 NFM, respectively, at 7 days post-feeding; moreover, according to the Fisher exact test, we observed significant differences in the mortality of PRMs fed the immune plasma against rFER2 PRM at 2–7 days post-feeding, and 3–7 days post-feeding in the immunized group of rFER2 TFM and rFER2 NFM, compared with those of the control group (Table 3). Kaplan–Meier curves revealed that the survival rate of PRMs fed with the immune plasma against each rFER2 was significantly lower than that of the control group (Figure 5A). Similar results were recorded in experiment 2. The survival rate of PRMs fed with immune plasmas against each rFER2 was significantly decreased (Figure 5B), and significant differences in the mortality rates were observed within 3–7 days post-feeding in the immunized groups of rFER2 PRM and rFER2 NFM and within 4–7 days post-feeding in the immunized group of rFER2 TFM (Table 4). Thus, the immune plasma against each rFER2 exhibited acaricidal effects on PRMs. Therefore, rFER2 could be a candidate antigen for the development of a universal vaccine across avian mites.

**Table 3:** Mortality of PRMs fed plasma from chickens immunized with recombinant ferritin 2 from different species of mites (experiment 1).

	**Days post-feeding**						
**Control group (*****n*** = **103)**							
No. of dead PRMs post-feeding	2	4	4	6	9	10	14
Mortality (%)	1.94	3.88	3.88	5.82	8.73	9.71	13.59
**Immunized group (rFER2 PRM**, ***n*** = **143)**							
No. of dead PRMs post-feeding	11	22	25	33	40	50	58
Mortality (%)	7.69	15.38	17.48	23.07	27.97	34.96	40.55
Chi-square	2.89	7.206	9.38	12.096	12.707	19.363	19.75
*P*-value	0.079	0.003^*^	1.06E-03^*^	1.77E-04^*^	1.67E-04^*^	4.3E-06^*^	4.12E-06^*^
Odds ratio	4.188	4.476	5.214	4.823	4.035	4.969	4.312
95% confidence interval	0.88–39.69	1.45–18.45	1.72–21.31	1.89–14.69	1.80–9.98	2.32–11.67	2.18–9.02
**Immunized group (rFER2 TFM**, ***n*** = **103)**							
No. of dead PRMs post-feeding	4	9	13	17	20	26	33
Mortality (%)	3.88	8.73	12.62	16.50	19.41	25.24	32.03
Chi-square	0.171	1.313	4.103	4.894	4.013	7.574	8.931
*P*-value	0.683	0.251	0.0401^*^	0.0252^*^	0.0437^*^	5.39E-03^*^	2.56E-03^*^
Odds ratio	2.033	2.36	3.554	3.178	2.505	3.123	2.981
95% confidence interval	0.28–22.96	0.63–10.85	1.05–15.51	1.13–10.31	1.02–6.61	1.36–7.73	1.42–6.52
**Immunized group (rFER2 NFM**, ***n*** = **61)**							
No. of dead PRMs post-feeding	2	7	10	13	17	21	24
Mortality (%)	3.27	11.47	16.39	21.31	27.87	34.42	39.34
Chi-square	1.6E-04	2.419	6.1603	7.521	9.125	13.699	37.437
*P*-value	0.629	0.102	8.35E-03^*^	4.56 E-03^*^	1.77 E-03^*^	1.56E-04^*^	6.18E-10^*^
Odds ratio	1.705	3.184	4.803	4.336	3.997	4.829	9.628
95% confidence interval	0.12–24.11	0.77–15.51	1.31–22.03	1.43–14.81	1.54–11.04	1.97–12.60	4.30–22.70

* *P* < 0.05 was considered statistically significant.

PRM, poultry red mite; TFM, tropical fowl mite; NFM, northern fowl mite.

**Table 4:** Mortality of PRMs fed plasma from chickens immunized with recombinant ferritin 2 from different species of mites (experiment 2).

	**Days post-feeding**						
	**1**	**2**	**3**	**4**	**5**	**6**	**7**
**Control group (*****n*** = **62)**							
No. of dead PRMs post-feeding	1	2	2	2	3	7	11
Mortality (%)	1.61	3.23	3.23	3.23	4.84	11.29	17.74
**Immunized group (rFER2 PRM**, ***n*** = **48)**							
No. of dead PRMs post-feeding	2	5	7	9	13	14	17
Mortality (%)	4.17	10.42	14.58	18.75	27.08	29.17	35.47
Chi-square	0.051	1.296	3.257	5.622	9.055	4.499	3.571
*P*-value	0.579	0.236	0.039^*^	0.0096^*^	0.0019^*^	0.027^*^	0.047^*^
Odds ratio	0.629	3.449	5.049	6.808	7.175	3.199	2.52
95% confidence interval	0.13–158.83	0.53–37.84	0.9–52.18	1.31–68.15	1.8–41.94	1.08–10.37	0.97–6.81
**Immunized group (rFER2 TFM**, ***n*** = **56)**							
No. of dead PRMs post-feeding	1	4	7	9	12	18	20
Mortality (%)	1.79	7.14	12.5	16.07	21.43	32.14	35.71
Chi-square	2.37E-31	0.299	2.396	4.325	5.879	6.464	0.023
*P*-value	1	0.421	0.0832	0.0243^*^	0.0109^*^	0.0069^*^	0.036^*^
Odds ratio	1.108	2.292	4.237	5.668	5.292	3.679	2.555
95% confidence interval	0.01–88.41	0.31–26.31	0.76–43.61	1.09–56.31	1.32–30.97	1.31–11.49	1.02–6.69
**Immunized group (rFER2 NFM**, ***n*** = **54)**							
No. of dead PRMs post-feeding	3	5	8	10	13	18	20
Mortality (%)	5.56	9.26	14.81	18.52	24.07	33.33	37.04
Chi-square	0.424	0.942	3.559	5.722	7.436	7.042	4.546
*P* value	0.337	0.248	0.0432^*^	0.0118^*^	0.0055^*^	0.00601^*^	0.0221^*^
Odds ratio	3.551	3.033	5.149	6.716	6.143	3.881	2.703
95% confidence interval	0.28–191.19	0.47–33.16	0.96–52.02	1.33–66.14	1.55–35.7	1.38–12.16	1.07–7.11

* *P* < 0.05 was considered statistically significant.

PRM, poultry red mite; TFM, tropical fowl mite; NFM, northern fowl mite.

## Discussion

Vaccine approaches have been focused on as a method for controlling PRMs to overcome the diminished effectiveness of acaricides and the selection of acaracide-resistant mites on poultry farms, in addition to their cost-effectiveness, low toxicity to the environment, and long-lasting action (42). However, other hematophagous mites, TFMs and NFMs, which are genetically similar to PRMs, cause problems similar to those of PRMs on poultry farms. Therefore, the development of a vaccine with broad protection efficacy against these avian mites could be a promising approach for their control in the poultry industry. The molecules involved in the critical physiological functions of avian mites are suitable vaccine antigens. FER2 is involved in iron transport and is critical for blood feeding and reproduction in ticks; moreover, FER2 is highly immunogenic and a useful candidate for anti-tick vaccines with broad protective efficacy across some tick species (27, 30, 43). FER2 has also been reported as a crucial molecule for the survival, reproduction, and blood digestion of PRMs. Moreover, acaricidal effects on PRMs by immunization have been demonstrated (25). Herein, we genetically identified and characterized *FER2* genes from TFMs and NFMs. In addition, the recombinant FER2 proteins from PRMs, TFMs, and NFMs were shown to be iron-binding proteins. Moreover, immune plasmas against each rFER2 showed cross-reactivity with rFER2 of different mites and acaricidal effects on PRMs, even when we used immune plasmas against rFER2 of TFMs and NFMs. Collectively, FER2 could be used as a vaccine antigen with protective efficacy against avian mites.

Genetic analysis showed that the *FER2* genes of PRMs, TFMs, and NFMs belonged to the cluster of secretory ferritins and were distinct from the cluster of intracellular ferritins. The secretory FER2 proteins of avian mites include signal peptides at the N-terminus. Similar to ferritins in vertebrates, ferritins of insects consist of H subunits containing ferroxidase centers (iron-binding sites) and light-chain (L) subunits containing amino acid residues with ferrihydrite nucleation centers (44). In this study, the ferroxidase centers of FER2 of avian mites were completely conserved, showing H-type subunits that are highly functional for catalytic activity in Fe(II) oxidation. We also observed the iron-binding abilities of rFER2-PRM, rFER2-TFM, and rFER2-NFM. Two types of ferritins have been identified in ticks (31, 45), and unlike FER1, FER2 has been recognized as a secretory protein in the tick hemolymph (29). Two types of ferritin have been identified in PRMs (25). The *FER2* gene of PRMs is expressed in all developmental stages, and RNAi analysis revealed critical functions for survival and reproduction (25). Unfortunately, in the present study, we could not elucidate the expression patterns of the *FER2* genes in the developmental stages of TFMs and NFMs and could not perform RNAi analysis on TFMs and NFMs because of the limited availability of TFM and NFM samples in Japan. However, we confirmed that the immune plasma against rFER2 of TFMs and NFMs cross-reacted with PRM rFER2 and exhibited anti-PRM effects. Collectively, these data suggest that the FER2 proteins identified in this study are secretory ferritins and have essential roles in the physiology of avian mites.

Host blood is an essential source of nutrients required for the growth and reproduction of hematophagous ectoparasites. During a single feed, a PRM can suck ~0.2 μL of the host blood (16) and is exposed to a large amount of iron. Excessive exposure to non-heme iron after hemoglobin digestion in midguts could be toxic to mites. Similar to the mechanisms in ticks (46), FERs are considered to play a role in iron homeostasis, although iron metabolism remains poorly understood in avian mites. According to a previous report, silencing of FER1 and FER2 affects feeding and oviposition in ticks, and FER2 depletion is linked to FER1 expression and altered iron homeostasis in ticks (31). Therefore, FER2 has been targeted as an effective vaccine candidate for ticks (27, 30, 43). Knockdown of FER1 and FER2 led to decreased blood digestion and oviposition, and increased mortality in PRMs; moreover, a significant increase in mortality of PRMs were recorded by immunization of chickens with rFER2 (rDg-FER1 in the original study) (25). In the present study, we observed the anti-PRM effects of rFER2 from TFMs and NFMs, and all immune plasmas cross-reacted with FER2 proteins of different mites. Therefore, these findings suggest the usefulness of FER2 as a vaccine antigen against TFMs and NFMs, and highlight FER2 as a candidate for the development of a universal vaccine against avian mites.

The development of a cross-protective vaccine for multi-tick species has been emphasized because of the non-uniform distribution of ticks worldwide. Cattle can get infected by various ticks due to their preferences, and the usefulness of common antigens with broad protective efficacy against different ticks has been reported (27, 30, 43, 47). Our research group has introduced a similar concept for avian mites, which poses a serious problem to the poultry industry. In addition to the development of anti-PRM vaccines, we are extending our work to determine the usefulness of anti-PRM vaccines for different avian mites. In this study, the immune plasma of chickens against each rFER2 cross-reacted with rFER2 proteins of different mites, and immune plasma against TFMs and NFMs showed acaricidal effects on PRMs by *in vitro* feeding. The development of a universal vaccine could be cost-effective for commercial production. The potential application of this kind of vaccine in poultry farms could prevent economic losses in production. However, there is a limitation in evaluating the acaricidal effects on TFMs and NFMs. To demonstrate the acaricidal effects of immune plasmas on TFMs and NFMs, *in vitro* assays using TFMs and NFMs for the assessment of vaccine antigens must be established, and challenge trials on chickens immunized with each mite are required to develop an effective universal vaccine for controlling avian hematophagous mites. Moreover, it is difficult to assess if immunization with rFER2 contributes to the improved economics based on the *in vitro* data; therefore, field trials are required to precisely evaluate the impact of vaccination with FER2. In addition, the search for other common antigens and the combined use of multiple antigens as a cocktail vaccine could further enhance the acaricidal effects on PRMs, TFMs, and NFMs.

## Conclusions

In the present study, we characterized FER2 from PRMs, TFMs, and NFMs and investigated its acaricidal effects as a vaccine antigen to assess the potential application of a universal vaccine across avian mites. The amino acid residues crucial for the oxidization of Fe(II) were conserved among the three species, and all rFER2 proteins tested showed iron-binding ability. Most importantly, the immune plasmas against rFER2 of PRMs, TFMs, and NFMs cross-reacted with rFER2 from different mites and exhibited acaricidal effects on PRMs, even in assays using immune plasmas against rFER2 of TFMs and NFMs. Thus, FER2 may be a useful vaccine antigen for avian mites. Further studies are needed to assess the usefulness of FER2 as a universal vaccine against avian mites.

## Data availability statement

The original contributions presented in the study are included in the article/Supplementary material, further inquiries can be directed to the corresponding author.

## Ethics statement

All animal experiments were performed in accordance with the guidelines and regulations of the Faculty of Veterinary Medicine, Hokkaido University, which is fully accredited by the Association for Assessment and Accreditation of Laboratory Animal Care International, and approved by the Institutional Animal Care and Use Committee of Hokkaido University (approval number: 20–0051).

## Author contributions

SW and SM conceived of and designed the study, analyzed the data, and drafted the manuscript. SW, SF, HS, JS, YM, LH, and SB conducted the experiments. SM, TO, NM, TS, EO, AT, SK, and KO provided intellectual input, laboratory materials, reagents, and analytical tools. All authors have reviewed and approved the final manuscript.
