# Supplementary material for: Potential of ferritin 2 as an antigen for the development of a universal vaccine for avian mites, poultry red mites, tropical fowl mites, and northern fowl mites
Source: Front Vet Sci. 2023 Apr 17;10:1182930. doi: 10.3389/fvets.2023.1182930 (PMC10149675; doi:10.3389/fvets.2023.1182930)
Supplement: Supplementary file 1 [file Data_Sheet_1.docx]

Supplementary Material

Potential of ferritin 2 as an antigen for the development of a universal vaccine for avian mites, poultry red mites, tropical fowl mites, and northern fowl mites

**Shwe Yee Win, Shiro Murata^*^, Sotaro Fujisawa, Hikari Seo, Jumpei Sato, Yoshinosuke Motai, Takumi Sato, Eiji Oishi, Akira Taneno, Lat Lat Htun, Saw Bawm, Tomohiro Okagawa, Naoya Maekawa, Satoru Konnai, Kazuhiko Ohashi**

*** Correspondence:** Shiro Murata: murata@vetmed.hokudai.ac.jp

# Supplementary Figures and Tables

## Supplementary Figures


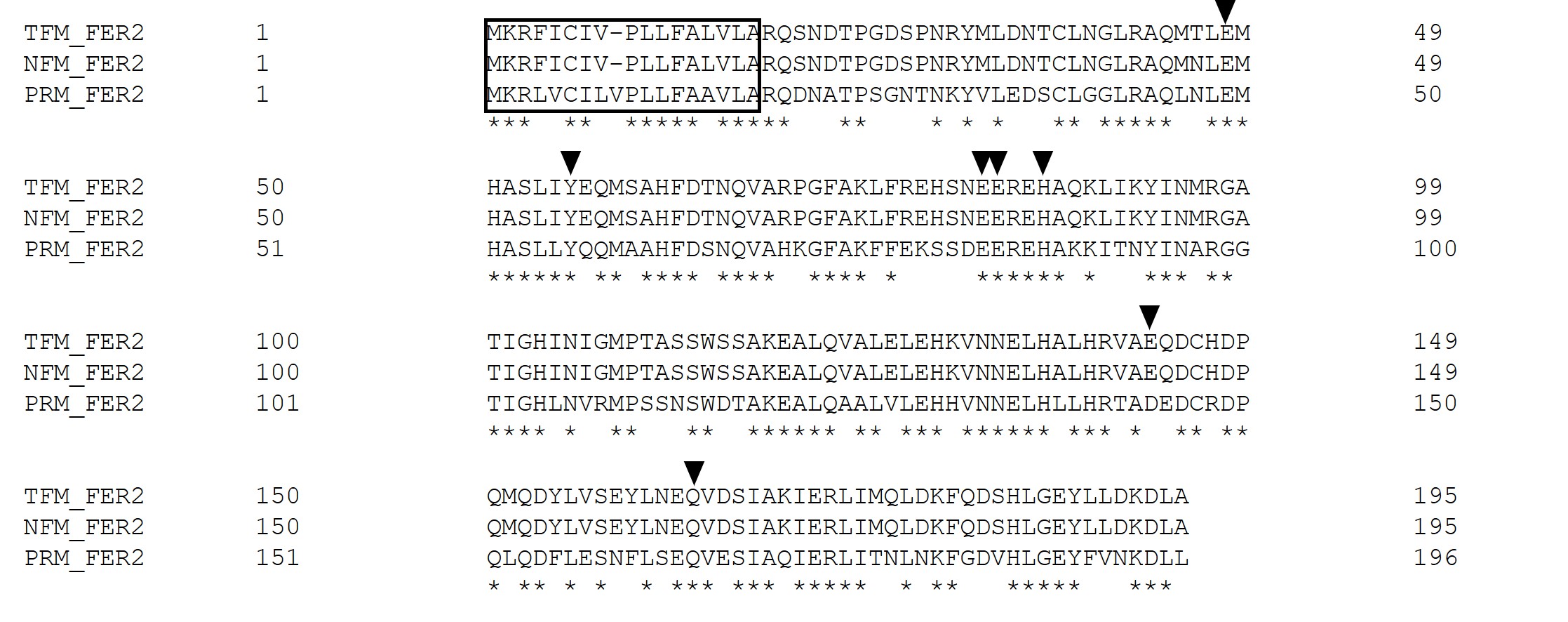


**Supplementary Figure 1.** The multiple alignment of ferritin 2 (FER2) of poultry red mites (PRMs), tropical fowl mites (TFMs), and northern fowl mites (NFMs). The deduced amino acid sequences of FER2 from TFMs and NFMs were aligned with that of PRM FER2. All FER2 proteins have the signal peptides (box) at the positions of 1–17 in TFMs and NFMs and 1–18 in PRMs. The black arrowheads indicate the ferroxidase centers of heavy chain subunits of FER2.


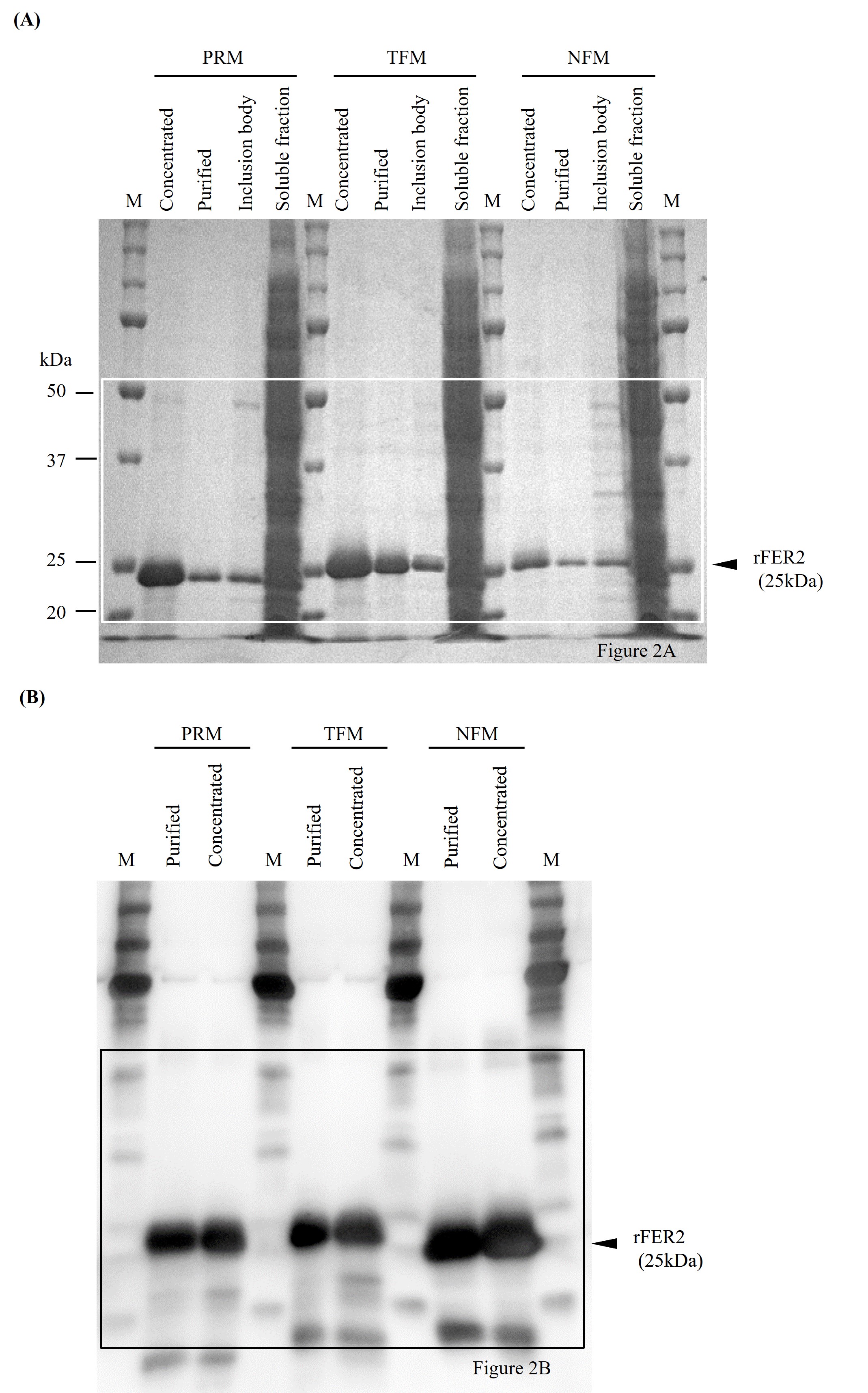


**Supplementary Figure 2.** Uncropped images for Figure 2. (A) SDS-PAGE analysis (B) Western blotting


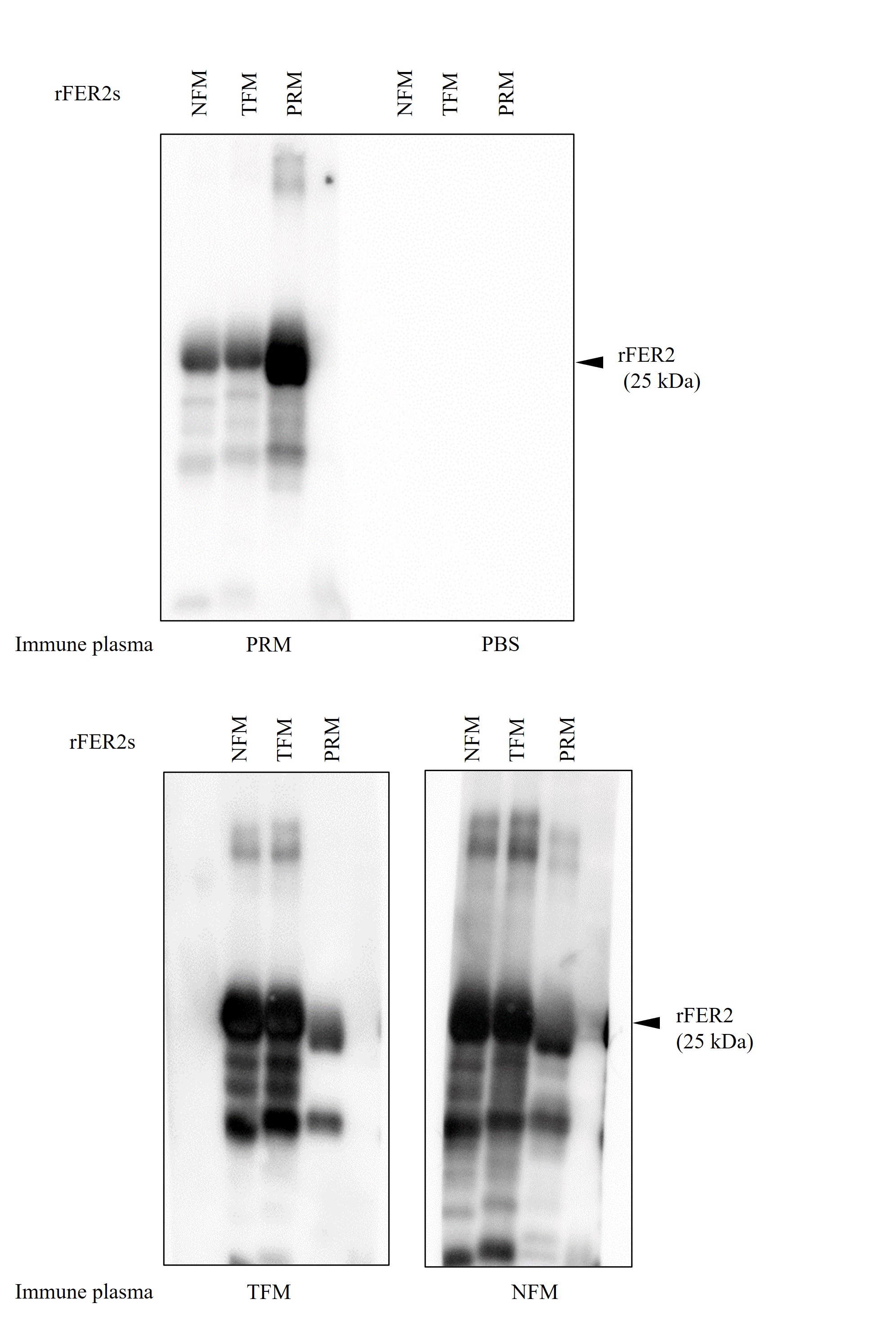


**Supplementary Figure 3.** Uncropped images for Figure 4.

## Supplementary Tables

| **Supplementary Table 1.** List of primers used for the amplification of *ferritin 2* genes in this study. | | | |
| --- | --- | --- | --- |
| Primer | Mite species | Intended use | Sequences (5´-3´) |
| FER2 outer -F | NFM & TFM | Partial gene amplification | TCGTRCCSCTGCTSTTCGC |
| FER2 outer- R |  |  | TCGGCATCCSDAYGTTGAKRT |
| FER2 inner -F |  |  | ACCCTCGARATGCAYGCYTC |
| FER2 inner-R |  |  | AGGWCWAGVGCRGCYTGVAG |
| GSP1 | NFM & TFM | 3′RACE | GTGAACACGCACAGAAGCTC |
| GSP2 |  |  | ATGTCTGCGCACTTTGACAC |
| GSP3 |  |  | GAGATGCACGCCTCTCTGAT |
| GSP1 | NFM & TFM | 5′RACE | CTCATTGTTGACTTTG |
| GSP2 |  |  | TGGCACTGCTCCACGAACTA |
| GSP3 |  |  | CTTGGCAAATCCTGGACGAG |
| rFER2 PRM-F | PRM | Construction for expression plasmids | AAGCATATGCGCCAAGATAATGCCACCCCATC |
| rFER2 PRM-R |  |  | ATCCTCGAGCTAGAGAAGGTCCTTGTTGACAAAGTATTCTCC |
| rFER2 TFM-F | NFM & TFM |  | AAGCATATGCGTCAGAGCAATGACACGCCT |
| rFER2 TFM-R |  |  | ATCCTCGAGCTAAGCGAGATCCTTGTCGAGGAGATA |
| *The NdeI and XhoI sites are underlined.  PRM, poultry red mite; NFM, northern fowl mite; TFM, tropical fowl mite; FER2, ferritin 2; rFER2, recombinant ferritin 2 | | | |

| **Supplementary Table 2.** List of *ferritin* genes used for phylogenetic analysis in Figure 1. | | | |
| --- | --- | --- | --- |
| Accession No. | GenBank | Product | Species |
| XM 003737350 | PREDICTED: *Metaseiulus occidentalis* ferritin | soma ferritin-like | *Metaseiulus occidentalis* |
| XM 003737351 | PREDICTED: *Metaseiulus occidentalis* ferritin | soma ferritin-like | *Metaseiulus occidentalis* |
| MNPL01001122 | *Tropilaelaps mercedesae* ferritin | ferritin | *Metaseiulus occidentalis* |
| HZ459285 | *Dermanyssus gallinae* ferritin 2 | ferritin 2 | *Dermanyssus gallinae* |
| MW798797 | *Dermanyssus gallinae* ferritin 2 | ferritin 2 | *Dermanyssus gallinae* |
| XM 022808087 | PREDICTED: *Varroa destructor* soma ferritin-like | soma ferritin-like | *Varroa destructor* |
| XM 022808086 | PREDICTED: *Varroa destructor* soma ferritin-like | soma ferritin-like | *Varroa destructor* |
| JQ922403 | *Rhipicephalus microplus* isolate Bhilwara ferritin 2 | ferritin 2 | *Rhipicephalus microplus* |
| MW346659 | *Dermacentor marginatus* isolate XJ-ZS-41 ferritin 2 | ferritin 2 | *Dermacentor marginatus* |
| MZ332506 | *Ornithodoros moubata* ferritin 2 | ferritin 2 | *Ornithodoros moubata* |
| EU885951 | *Ixodes ricinus* secreted ferritin | secreted ferritin | *Ixodes ricinus* |
| KF311110 | *Ixodes persulcatus* ferritin 2 | ferritin 2 | *Ixodes persulcatus* |
| XM 029988441 | PREDICTED: *Ixodes scapularis* ferritin heavy chain | ferritin heavy chain | *Ixodes scapularis* |
| MW798798 | *Dermanyssus gallinae* ferritin 1 mRNA | ferritin 1 mRNA | *Dermanyssus gallinae* |
| XM 022787705 | PREDICTED: *Varroa destructor* soma ferritin-like | soma ferritin-like | *Varroa destructor* |
| XM 018638352 | PREDICTED: *Metaseiulus occidentalis* ferritin heavy chain | ferritin heavy chain | *Metaseiulus occidentalis* |
| XM 050182915 | PREDICTED: *Dermacentor andersoni* soma ferritin-like | soma ferritin-like | *Dermacentor andersoni* |
| AY277902 | *Boophilus microplus* ferritin mRNA | ferritin mRNA | *Boophilus microplus* |
| MN937442 | *Haemaphysalis flava* ferritin 1 mRNA | ferritin 1 mRNA | *Haemaphysalis flava* |
| AY277905 | *Haemaphysalis longicornis* ferritin mRNA | ferritin mRNA | *Haemaphysalis longicornis* |
| BM486522 | *Gallus gallus* ferritin | ferritin | *Gallus gallus* |
